# 

**Published:** 1924-09

**Authors:** 


### HOSPITAL and HEALTH REVIEW

*Established 1886* *No.* 1878. *Vol. LXXIII*

*New Series.* Vol. III. No. 36. *September,* 1924. Price Sixpence

CONTENTS

| | PAGE |
|---|---|
| Longevity and Disability | 259 |
| When Labour Moves | 260 |
| Notes and Comments | 261 |
| Neither Dead nor Dying—The New Housing Act — Preserving Food — Broadcasting Health Talks—The Washington Maternity Convention—Jerusalem and Geneva—Impecunious State Hospitals—The Smoke Abatement Bill — "For Remembrance" — The Latest Profession—The Fetish of Free Choice of Doctor—Too Much Medicine—Warming the House—Hay Fever and Glass Cases—Milk from Tuberculous Cows | |
| Hospital Men of Mark: Lord Armstrong and Mr. S. Dunstan | 265 |
| Making a Practice. By A Doctor's Wife | 266 |
| Clean Milk Without Expense | 267 |
| First Aid for the Hop-Picker | 268 |
| The Tuberculous Child. By C. Lee Pattison, M.R.C.S., L.R.C.P. | 269 |
| Drugs from Venezuela | 270 |
| The Comradeship of Science | 271 |
| Heartbeats by Radio | 272 |
| Medical Service Afloat | 274 |
| Ipswich Hospital's New Memorial Wing | 274 |
| The Public Health: Interviews with Local Authorities: Marylebone | 275 |
| A Medical School Library | 278 |
| New Medical Schools | 278 |
| Hospital Progress and Finance | 279 |
| Reviews of Books | 281 |
| The Medical Curriculum | 284 |
| Echoes of the Month | 290 |
| Hospital and Health News | 291 |
| Recent Appointments | 292 |
| Answers to Correspondents | 292 |

